# Cardiovascular risk factors in pre-pubertal schoolchildren in Angola

**DOI:** 10.5830/CVJA-2016-029

**Published:** 2016

**Authors:** Amílcar BT Silva, Sérgio L Rodrigues, Marcelo P Baldo, José Geraldo Mill, Amílcar BT Silva, Daniel P Capingana, Pedro Magalhães, Mauer AA Gonçalves, Miguel SB Mateus, Maria del Carmen B Molina

**Affiliations:** Department of Physiological Sciences, Federal University of Espírito Santo, Espírito Santo, Brazil; Department of Physiological Sciences, Federal University of Espírito Santo, Espírito Santo, Brazil; Department of Physiological Sciences, Federal University of Espírito Santo, Espírito Santo, Brazil; Department of Physiological Sciences, Federal University of Espírito Santo, Espírito Santo, Brazil; Department of Physiological Sciences, School of Medicine, Agostinho Neto University, Luanda, Angola; Department of Physiological Sciences, School of Medicine, Agostinho Neto University, Luanda, Angola; Department of Physiological Sciences, School of Medicine, Agostinho Neto University, Luanda, Angola; Department of Physiological Sciences, School of Medicine, Agostinho Neto University, Luanda, Angola; Department of Physiological Sciences, School of Medicine, Agostinho Neto University, Luanda, Angola; Department of Public Health, Federal University of Espírito Santo, Espírito Santo, Brazil

**Keywords:** obesity, overweight, childern, blood pressure, cardiovascular risk

## Abstract

**Methods:**

The incidence of obesity is increasing worldwide, especially in countries with accelerated economic growth. We determined the prevalence of and associations between overweight/ obesity and cardiovascular risk factors in pre-pubertal (seven- to 11-year-old) schoolchildren (both genders, n = 198) in Luanda, Angola. Biochemical (fasting blood) and clinical examinations were obtained in a single visit. Data are reported as prevalence (95% confidence intervals) and association (r, Pearson).

**Results:**

Prevalence of overweight/obesity was 17.7% (12.4–23.0%), high blood pressure (BP < 90% percentile) was 14.6% (9.7–19.5%), elevated glucose level was 16.7% (11.5–21.9%) and total cholesterol level < 170 mg/dl (4.4 mmol/l) was 69.2% (62.8–75.6%). Significant associations between body mass index (BMI) and systolic and diastolic BP (r = 0.46 and 0.40, respectively; p < 0.05) were found. No association between BMI and elevated glucose or cholesterol levels was found.

**Conclusion:**

The prevalence of cardiovascular risk factors was high in pre-pubertal schoolchildren in Angola and fat accumulation was directly associated with blood pressure increase but not with other cardiovascular risk factors.

## Abstract

Cardiovascular disease (CVD) is the leading cause of death worldwide. Although its most overt manifestations, such as hypertension, myocardial infarction and stroke, appear more frequently in adulthood, there is growing evidence that the risk factors for these events can appear precociously in the course of life, even in childhood.1-3 Some studies suggest that the pathophysiological processes leading to the onset of CVD in earlier phases of adult life could be detected as early as the foetal period.^4^,^6^

Hypertension and obesity are two cardiovascular risk factors with a high prevalence in the adult population in virtually all countries. Their incidence is occurring at increasingly younger ages; today, these conditions affect a significant proportion of children and adolescents.^7^,^9^ Therefore early evaluation of predisposing factors for these disorders, such as low birth weight10 and inadequate diet,^11^,^12^ may contribute to the adoption of early interventions for CVD prevention in adulthood.^13^

Obesity in adults has become a global pandemic, affecting both developed and developing countries.^14^ Given that the accumulation of body fat is due in large part to the adoption of inadequate dietary and lifestyle habits, it appears that the prevalence of obesity is increasing systematically in children and adolescents around the world.

Following the overweight and obesity pandemic, an increased incidence of hypertension, type 2 diabetes and dyslipidaemia is to be expected.^2^,8 Dyslipidaemia, in parallel with insulin resistance, is one of the most important risk factors for the onset and progression of atherosclerosis, and probably has a direct relationship with obesity because both processes involve similar lifestyle predictors.^14^

The prevalence of dyslipidaemia in children and adolescents worldwide varies between 2.9 and 33%, depending on the cut-off points used and the age range of individuals included in the study.^15^ However, there is now strong evidence of an increased incidence of these conditions in the pre- and post-pubertal periods.^16^,^18^ Most studies, however, have been conducted in developed countries with few studies performed in Africa to date.^19^

Obesity and cardiovascular risk factors tend to increase in countries with rapid economic and social transformation. Angola is an African country that has experienced rapid economic growth over the last 15 years, following the end of the civil war. However, no existing studies have examined cardiovascular risk in Angolan children. Therefore the aim of this study was to determine the prevalence and severity of cardiovascular risk factors in pre-pubertal schoolchildren in Luanda, Angola, and to determine the contribution of excessive body weight to these findings.

## Methods

A cross-sectional, observational and descriptive study was conducted in a sample of children enrolled in the first cycle of primary education in a public school in Luanda, the Angolan capital. The project was carried out after obtaining authorisation from the Provincial Directorate of Education, the Education Chamber of Rangel municipality and the directors of the school 5008 (Our Lady of Light).

Consent for the involvement of the community in the study was obtained in meetings with the school staff, parents and/or guardians of the children and the children to clarify the study purposes and methods. The project was approved by the Independent Ethics Committee on Research of the Faculty of Medicine of Agostinho Neto University, following the standards procedures in human research in accordance with the Declaration of Helsinki.

In the 2011 academic year, 1 015 students (aged five to 12 years) were enrolled in the Our Lady of Light school and 719 (70.8%) were eligible for the study (seven to 11 years). This school was chosen because it is near the Faculty of Medicine, where data were collected and because it is located in a neighborhood with most of the families belonging to the middle class.

After meeting with parents or guardians, we sent an envelope containing an invitation letter and a questionnaire to a random subsample of 290 students. The questionnaire required completion for information on the conditions of birth and the child’s life, including pregnancy history, birth weight, period of exclusive breastfeeding, self-reported diseases, and sociodemographic information of the family. Information about birth weight and the duration of exclusive breastfeeding in addition to signing of the consent form were necessary for final inclusion in the project.

Of the 290 questionnaires distributed, 248 (85.5%) were returned. Eleven children were excluded due to lack of essential information. Of the 237 selected, 23 did not appear at the venue for the examination. The set of examinations to investigate the presence of cardiovascular risk factors was performed on 214 children.

All children were classified according to the Tanner scale,^20^ obtained by self-evaluation of genitalia and breast development. According to this procedure, 16 children were excluded from our analysis because they were in Tanner stage II, or because they had completed 12 years between enrolment and the examination. Therefore, the data in this study refer to 198 pre-pubertal (Tanner stage I) and apparently healthy schoolchildren.

All study participants presented to the Laboratory of Functional Tests and Physiology of the Faculty of Medicine on a pre-scheduled morning in a fasting condition (10–14 hours). Examinations were performed from June 2012 to November 2013. A previous pilot study (March 2012) was performed on 30 children (not included in this study) to standardise the protocol and to train examiners. Sociodemographic data (age, gender, race, school grade), physical activity and dietary habits were obtained in an interview with the child and the mother or guardian.

Blood was collected by venipuncture of the forearm and processed on the same day by the Department of Biochemistry (Faculty of Medicine) to determine glucose, urea, uric acid, creatinine, total cholesterol, high-density lipoprotein (HDL) cholesterol and triglyceride levels. Only reagents of BioSystems SA (Barcelona, Spain) were used. Low-density lipoprotein (LDL) cholesterol concentration was calculated by the Friedewald formula for triglycerides < 400 mg/dl (4.52 mmol/l).

Biochemical variables in the blood were classified according to the criteria established by the American Academy of Pediatrics and the American Heart Association (American Guidelines for Cardiovascular Health and Risk Reduction in Children and Adolescents 2012).^21^ In brief, the lipid profile was classified as follows:

total cholesterol < 170 mg/d (< 4.4 mmol/l): acceptable; 170–199 mg/dl (5.15 mmol/l): borderline; ≥ 200 mg/dl (≥ 5.18 mmol/l): high
LDL cholesterol < 110 mg/dl (< 2.85 mmol/l): acceptable; 110–129 mg/dl (2.85–3.34 mmol/l): borderline; and ≥ 130 mg/ dl (≥ 3.37 mmol/l): high
triglycerides (0–9 years) < 75 mg/dl (< 0.85 mmol/l): acceptable; 75–99 mg/dl (0.85–1.12 mmol/l): borderline; ≥ 100 mg/dl (≥ 1.13 mmol/l): high
triglycerides (10–19 years) < 90 mg/dl (< 1.02 mmol/l): acceptable; 90–129 mg/dl (1.02–1.46 mmol/l): borderline; and ≥ 130 mg/dl (≥ 1.47 mmol/l): high
HDL cholesterol < 45 mg/dl (< 1.17 mmol/l): acceptable; 40–45 mg/dl (1.04–1.17 mmol/l): borderline; and < 40 mg/dl (< 1.04 mmol/l): low.^21^

According to fasting blood glucose criteria, the children were classified as normoglycaemic (60–99 mg/dl) (3.33–5.49 mmol/l), glucose intolerant (100–125 mg/dl) (5.55–6.94 mmol/l) or hyperglycaemic (< 125 mg/dl) (< 6.94 mmol/l). No child was on treatment with insulin or oral hypoglycaemic agents.

Body weight was obtained using a digital electronic scale (SECA, Mod 763, Germany) with 0.1-kg precision while fasting and after voiding. Children were barefoot and clothed with undergarments only. Height was measured using a fixed stadiometer with 0.5-cm precision. The child was placed on the central part of the scale platform with heels together, head and buttocks resting against the stadiometer and eyes looking toward the Frankfurt horizontal plane.

The body mass index (BMI) was calculated by dividing body weight by height squared (kg/m^2^). Percentiles (P) of height, weight and BMI of each child were calculated as presented in https://www.bcm.edu/bodycomplab/.^22^ BMI classification was obtained according to the following World Health Organisation (WHO) parameters: underweight (BMI < P5), normal (BMI ≥ P5 and < P85), overweight (BMI ≥ P85 and < P95) and obese (BMI ≥ P95).^23^

The left arm circumference was obtained with an inelastic tape at the midpoint between the acromion (proximal) and olecranon process (distal). The waist (WC) and hip circumferences (HC) were measured with an inelastic tape with the child in a standing position and at the end-expiration phase. Measurements (0.1- mm precision) were obtained in front of a mirror to facilitate the tape positioning in the horizontal plane. WC was measured at the midpoint between the lower edge of the rib cage and the anterior superior iliac crest. HC was measured at the level of the great trochanter, circling the hip on the most prominent point between the waist and the thigh. The waist-to-hip ratio (WHR) was obtained by dividing WC by HC. The thickness of four skinfolds (triceps, supra-iliac, subscapular and abdominal) was measured with a manual caliper (Sanny) with a precision of 1 mm.

Body composition was obtained by tetrapolar bio-impedance with the Maltron BioScan Analyser (Model 916, Maltron Int Ltd, UK). Two electrodes were placed on the dorsal surface of the radiocarpal joint and metacarpal of the right hand and the other two on the dorsal surface of the right foot (tibiotarsal and metatarsal joint region) according to the manufacturer’s instructions. Body fat and percentage of body fat were used to indicate overall obesity.

Blood pressure was measured with an automatic sphygmomanometer (OMRON®, Model HEM-742 IntelliSense INT, China) in the sitting position in a comfortable room and after bladder emptying. The cuff size was chosen according to the circumference of the child’s arm and the manufacturer’s recommendation. Three measurements were taken on the left arm at two-minute intervals after a five- to 10-minute resting period. The forearm was supported on a flat surface at nearly 120° with the arm.

Resting systolic (SBP) and diastolic blood pressure (DBP) and heart rate (HR) were determined as the arithmetic mean of the last two measures. Pulse pressure (PP) was calculated as the difference between SBP and DBP. The mean arterial pressure (MAP) was calculated from the formula [SBP + (2 × DBP)]/3. The blood pressure percentile was determined using available software (https://www.bcm.edu/bodycomplab/), which accounts for the gender, height and age of each child.

Blood pressure was classified according to the WHO criteria.^23^ Briefly, it was considered normal if the SBP and DBP were below the 90th percentile. If SBP or DBP were above the 95th percentile (P95), the child was included in the category of ‘high BP’. If the SBP or DBP was intermediary between the extremes (< P90 < P95), the child was classified as ‘borderline BP’.

## Statistical analysis

Continuous variables are expressed as mean ± standard deviation when normally distributed or as median and interquartile range when the normal distribution model was not accepted (Kolmogorov–Smirnov test). Comparison of two means was done with the Student’s t-test for normal variables, and comparison of two medians was performed with the Wilkinson test. The comparison of proportions of categorical variables in two or more groups was performed using the chi-squared test. Comparison of means in three or more groups was performed by one-way ANOVA followed by the post hoc Tukey’s test. The degree of association between continuous variables was obtained with Pearson’s correlation coefficient (r).

A multivariate analysis (stepwise forward procedure) was used to indicate independent predictors of systolic and diastolic blood pressure. Gender, birth weight, age, height, body weight, body mass index, absolute and relative fat mass and lean mass (all with p < 0.05 in bivariate analysis) were included in the model as predictors. Co-linearity variables were automatically excluded from the model. Statistical analyses were performed using SPSS for Windows, version 20.0. The significance level for all tests was set at p < 5%.

## Results

Table 1 shows the main clinical characteristics of the sample, divided by gender. There was a predominance of girls (61.1%) and, as expected, children of black race (95.5%). Only nine children showed intermediate skin colour, suggesting a mixed ancestry of black and white. The boys had an overall birth weight higher than that of the girls, and a low birth weight (< 2 500 g) was reported in only 15 children.

**Table 1:** Physical and clinical characteristics of pre-pubertal schoolchildren in Luanda in 2012

*Variables*	*Boys (n = 77)*	*Girls (n = 121)*	*All (n = 198)*	*p-value*
Age (years)	9.43 ± 1.03	9.21 ± 1.12	9.29 ± 1.41	0.18
Birth weight (kg)	3.29 ± 0.56	3.12 ± 0.58	3.19 ± 0.58	0.05
Current weight (kg)	33.08 ± 9.06	33.07 ± 10.2	33.07 ± 9.72	0.99
Height (cm)	136.7 ± 8.05	137.8 ± 9.75	137.4 ± 9.12	0.44
BMI (kg/m^2^)	17.70 ± 4.0	17.19 ± 3.61	17.39 ± 3.76	0.36
WC (cm)	59.58 ± 8.94	58.38 ± 9.25	58.85 ± 9.13	0.37
HC (cm)	71.82 ± 9.71	71.95 ± 10.4	71.9 ± 10.12	0.93
WHR	0.82 ± 0.04	0.81 ± 0.04	0.81 ± 0.03	0.008
Fat mass (kg)	6.36 ± 3.97	6.85 ± 4.14	6.66 ± 4.07	0.41
SBP (mmHg)	104.7 ± 8.9	103.8 ± 8.1	104.1 ± 4.2	0.46
DBP (mmHg)	62.6 ± 7.8	63.6 ± 6.3	63.2 ± 0.4	0.33
HR (bpm)	80.1 ± 9.5	84.7 ± 10.2	82.9 ± 13.4	0.002
Glycaemia (mg/dl)	87.4 ± 15.3	86.5 ± 14.4	86.8 ± 15.5	0.69
(mmol/l)	4.85 ± 0.85	4.80 ± 0.80	4.82 ± 0.86	
TC (mg/dl)	170.9 ± 36.8	172.5 ± 34.4	172.5 ± 34.4.1	0.75
(mmol/l)	4.43 ± 0.95	4.47 ± 0.89	4.45 ± 0.88	
Triglycerides (mg/dl)	66.3 ± 31.9	63.5 ± 29.4	64.6 ± 4.0	0.54
(mmol/l)	0.75 ± 0.36	0.72 ± 0.33	0.73 ± 0.05	
LDL-C (mg/dl)	102.1 ± 33.2	101.4 ± 33.2	101.7 ± 22.6	0.89
(mmol/l)	2.64 ± 0.86	2.63 ± 0.86	2.63 ± 0.59	
HDL-C (mg/dl)	56.8 ± 13.1	59.5 ± 12.3	58.5 ± 9.1	0.13
(mmol/l)	1.47 ± 0.34	1.54 ± 0.32	1.52 ± 0.24	LDL-C/HDL-C
LDL-C/HDL-C	1.9 ± 0.8	1.9 ± 0.8	1.8 ± 0.3	0.28

Data presented as mean ± standard deviation, BMI; body mass index, WC, waist circumference; HC, hip circumference; WHR, waist/hip ratio, SBP; systolic blood pressure; DBP; diastolic blood pressure, HR; heart rate, TC, total cholesterol; LDL-C; low-density lipoprotein cholesterol, HDL-C; high-density lipoprotein cholesterol.

It can be observed (Table 1) that most of the anthropometric and biochemical variables were similar in pre-pubertal boys and girls, with the exception of HR, which was higher in girls, and WHR, which was higher in boys. According to BMI classification, excessive body weight was found in 17.7% of the sample (95% CI = 12.4–23.0%), with 7.1% being overweight and 10.6% obese. Eleven children were underweight and the remaining 152 (76.7%) children were of normal weight.

Table 2 shows the anthropometric, haemodynamic and biochemical variables according to BMI classification. Age across the four groups was similar (p > 0.05). As expected, the current body weight of the overweight and obese groups was higher than that of the normal group. The current weight was 61% higher in the obese group than in the normal group. Interestingly, birth weight was also higher in the groups with excessive fat mass. As expected, other anthropometric variables related to fat accumulation were also different between the normal BMI group and the groups with excessive fat accumulation. The obese group showed 2.56 times more fat mass than the normal BMI group.

**Table 2:** Physical and clinical characteristics of pre-pubertal schoolchildren according to body mass index classification

**	*Underweight*	*Normal*	*OverWeight*	**Obese	
*BMI class*	*(n = 11)*	*(n = 152)*	*(n = 14)*	*(n = 21)*	*p-value*
Age (years)	9.6 ± 1.1	9.2 ± 1.0	9.6 ± 1.3	9.4 ± 1.4	0.707
Birth weight (kg)	3.27 ± 0.6	3.12 ± 0.6	3.46 ± 0.4	3.47 ± 0.4^#^	0.046
Current weight (kg)	25.6 ± 6.6	30.1 ± 5.1	42.3 ± 8.8^#^	52.1 ± 11.1^#§^	< 0.001
Height (cm)	137.2 ± 15.4	136.4 ± 8.3	141.8 ± 10.8	141.7 ± 7.7^#^	0.054
BMI (kg/m^2^)	13.3 ± 0.8^#^	16.2 ± 1.4	20.8 ± 1.4#^#^	26.2 ± 3.6^#§^	< 0.001
WC (cm)	50.6 ± 4.7^#^	55.9 ± 4.7	68.1 ± 5.8^#^	78.5 ± 1.7^#^	< 0.001
HC (cm)	62.4 ± 6.4^#^	68.8 ± 6.1	83.7 ± 6.1^#^	91.3 ± 7.8	< 0.001
WHR	0.81 ± 0.04	0.81 ± 0.04	0.81 ± 0.03	0.86 ± 0.03^#§^	< 0.001
Fat mass (kg)	3.6 ± 0.7	5.3 ± 1.8	10.5 ± 3.8^#^	15.3 ± 4.6^#§^	< 0.001
SBP (mmHg)	99.0 ± 8.9	103.1 ± 7.6	106.1 ± 6.7	113.1 ± 8.9^#§^	< 0.001
DBP (mmHg)	60.7 ± 3.3	62.2 ± 6.4	65.3 ± 7.2	70.1 ± 7.5^#^	< 0.001
HR (bpm)	85.5 ± 10.3	83.2 ± 10	81.4 ± 12.5	80.2 ± 9.7	0.585
Glycaemia (mg/dl)	91.3 ± 13.4	87.5 ± 14.5	83.7 ± 17.8	82.0 ± 14.8	0.327
(mmol/l)	5.07 ± 0.74	4.86 ± 0.80	4.65 ± 0.99	4.55 ± 0.82	
TC (mg/dl)	194.6 ± 40.9	170.6 ± 34.5	176.3 ± 35.3	166.3 ± 35.5	0.127
(mmol/l)	5.04 ± 1.06	4.42 ± 0.89	4.57 ± 0.91	4.31 ± 0.92	
Triglycerides (mg/dl)	75.8 ± 26.7	63.4 ± 30.1	49.7 ± 17.9	77.5 ± 35.7^§^	0.039
(mmol/l)	0.86 ± 0.30	0.72 ± 0.34	0.56 ± 0.20	0.88 ± 0.40	
LDL-C (mg/dl)	115.6 ± 40.3	105.5 ± 32.4	109.4 ± 32.3	97.5 ± 34.6	0.458
(mmol/l)	2.99 ± 1.04	2.73 ± 0.84	2.83 ± 0.84	2.53 ± 0.90	
HDL-C (mg/dl)	63.5 ± 9.89	58.7 ± 13.1	56.5 ± 8.67	54.9 ± 12.5	0.256
(mmol/l)	1.64 ± 0.26	1.52 ± 0.34	1.46 ± 0.22	1.42 ± 0.32	
LDL-C/HDL-C	1.83 ± 0.7	1.82 ± 0.7	1.97 ± 0.6	1.89 ± 0.91	0.973

Data presented as mean ± standard deviation, BMI; body mass index, WC, waist circumference; HC, hip circumference; WHR, waist/hip ratio, SBP; systolic blood pressure; DBP; diastolic blood pressure, HR; heart rate, TC, total cholesterol; LDL-C; low-density lipoprotein cholesterol, HDL-C; high-density lipoprotein cholesterol.

^#^Difference between normal BMI with underweight, overweight and obesity;

^§^Difference between overweight and obesity.

While all biochemical variables were unaffected along BMI categories, except for triglycerides, which showed higher values in the obese group, a different pattern was observed in relation to blood pressure. An obvious gradient of increasing SBP and DBP values was observed from the underweight group to the obese group. SBP was statistically higher in the obese group compared with the underweight, normal and overweight groups, while the DBP was significantly different between the obese groups and the normal and underweight groups.

Significant differences among other variables are shown in Table 2. Comparisons were performed between the normal-weight group and the other three groups, and between the overweight and the obese groups only.

Prevalence of cardiovascular risk factors according to BMI classification is shown in Table 3. No relationship between fat accumulation and the presence of risk factors was observed in this age group. Moreover, prevalence of risk factors was similar in both genders (Table 4).

**Table 3:** Prevalence of cardiovascular risk factors in pre-pubertal schoolchildren according to presence of overweight or obesity (high weight group)

**	*Body weight*				
	*Normal*		*High*		
*Variable*	*n*	*%*	*n*	*%*	*p-value*
Prehypertension	13	7.97	7	20	0.066
Hypertension	6	3.1	3	8.6	0.416
Glucose intolerance	28	17.2	5	14.3	0.868
High TC (≥ 170 mg/dl) (≥ 4.4 mmol/l)	82	50.3	17	48.6	0.998
High LDL-C (≥ 110 mg/dl) (≥ 2.85 mmol/l)	67	41.1	15	42.8	0.998
Low HDL-C (< 45 mg/dl) (< 1.17 mmol/l)	16	9.8	3	8.6	0.929
High triglycerides	44	26.9	10	28.6	0.985
FHH	111	68.1	21	60	0.469
					

TC: total cholesterol, LDL-C: low-density lipoprotein cholesterol, HDL-C: highdensity lipoprotein cholesterol, FHH: family history of hypertension.

**Table 4:** Prevalence of cardiovascular risk factors by gender

	*Boys*		*Girls*		
*CV risk factors*	*n*	*%*	*n*	*%*	*p-value*
Overweight	5	6.5	9	7.4	0.975
Obese	9	11.7	12	9.9	0.875
Prehypertension	8	10.4	12	9.9	0.893
Hypertension	4	5.2	5	4.1	0.996
Glucose intolerance	15	19.5	18	14.9	0.514
High TC (≥ 170 mg/dl) (≥ 4.4 mmol/l)	34	44.1	65	53.7	0.243
High LDL-C (≥ 110 mg/dl) (≥ 2.85 mmol/l)	32	41.5	49	40.5	0.998
Low HDL-C (< 45 mg/dl) (< 1.17 mmol/l)	14	18.2	5	4.1	0.002
High triglycerides	22	28.6	32	26.4	0.870
FHH	56	72.7	77	63.6	0.241

CV: cardiovascular, TC: total cholesterol, LDL-C; low-density lipoprotein cholesterol, HDL-C: high-density lipoprotein cholesterol, FHH: family history of hypertension.

Normal blood pressure values were found in 166 (83.8%) children and elevated values were found in 29 (14.6%) children. Simultaneous elevation of both SBP and DBP was found in five children, while 16 showed an increase of only SBP and eight showed an increase of only of DBP. Hypertension was detected in nine children and pre-hypertension in 20, without significant difference between genders (prevalence of 3.5 and 11.1%, respectively). Positive familial history of hypertension was identified in 132 (66.7%) children. However, this trait was not associated with the presence of elevated blood pressure in the studied population (χ2 = 0.247, p = 0.618).

Another cardiovascular risk factor investigated was dyslipidaemia. The frequency of this condition was quite high, with 69.2% of the sample having at least one lipid value out of the normal range for this age group. This finding was unrelated to gender (71.4% for boys vs 67.8% for girls, p = 0.70) and BMI. The most common change in lipid profile was the presence of high total cholesterol levels, followed by an increase in LDL cholesterol and triglyceride levels. Low HDL cholesterol level was found in 9.1% of children, without a significant difference between genders.

Table 5 shows the correlations between the three indices of excessive fat accumulation and birth weight and cardiovascular risk factors. Either SBP or DBP were moderately associated with fat accumulation. This association was significant independent of global (BMI and % fat) or central (WC) obesity indices. However, no significant association was observed in our study between birth weight and blood pressure or other cardiovascular risk factors.

**Table 5:** Correlation coefficient between age, anthropometric variable and birth weight in cardiovascular risk factors in pre-pubertal schoolchildren

**	BMI**		*%fat*		*WC*		*Age*	
*Variable*	*r*	*p*	*r*	*p*	*r*	*p*	*r*	*p*
Glycaemia	-0.146	0.02	-0.130	0.034	–0.095	0.092	0.000	0.50
TC	–0.099	0.083	–0.006	0.136	–0.095	0.091	0.017	0.41
Triglycerides	0.086	0.115	0.081	0.129	0.143	0.022	0.111	0.60
HDL-C	-0.16	0.014	–0.109	0.064	–0.142	0.023	–0.02	0.40
SBP	0.460	< 0.05	0.370	< 0.05	0.462	< 0.05	0.20	0.02
DBP	0.401	< 0.05	0.432	< 0.05	0.416	< 0.05	0.128	0.03
Birth weight	0.169	0.009	0.208	0.002	0.193	0.003	-0.015	0.42

r: Pearson correlation coefficient; BMI: body mass index; SBP: systolic blood pressure; DBP: diastolic blood pressure; TC, total cholesterol; HDL-C: highdensity lipoprotein cholesterol; WC: waist circumference.

An inverse correlation between fat accumulation and HDL cholesterol level and glycaemia was also observed. In a multivariate linear regression, we observed that body weight (kg) was the only independent predictor of SBP (SBP = 0.402 × body weight + 90.8) while the fat mass (kg) was the only independent predictor of DBP (DBP = 0.759 × fat mass + 58.1).

## Discussion

The increased incidence of CVD risk factors is a growing reality in children and adolescents, according to studies in several countries.^1^,^16^,^18^,^24^,^29^ Few studies, however, have been carried out in African countries. Our study aimed to characterise the current prevalence and severity of CVD risk factors in pre-pubertal schoolchildren living in Luanda, Angola. Angola has experienced rapid economic growth in this century and as observed in other countries, this fact should translate into a rapid epidemiological transition affecting the whole population. Studies on this topic are inadequate in the Angolan population.

Our study shows that the prevalence of overweight and obesity was approximately 14% in the studied age group and that fat accumulation correlated with high blood pressure values. More importantly, we also showed an unexpected elevated prevalence of dyslipidaemia (mainly high total cholesterol levels), which, according to our data, was not associated with fat accumulation.

Similar to other studies in pre-pubertal children^16^,^25^,^26^ no significant difference was found between boys and girls regarding physical and clinical characteristics, indicating that such differences will appear later on during puberty. Our study was performed in pre-pubertal children only, to avoid the confounding influence of sex hormones and fat accumulation on blood pressure and other cardiovascular risk factors.

It was possible to confirm in this group of children that borderline or high blood pressure values were associated with fat accumulation, without significant differences if overall (BMI, % fat) or central obesity indices (WC) were considered. Some studies show a stronger association between DBP and central obesity. However, the small sample size and the relatively small number of obese and hypertensive children may have hampered such analysis.

The prevalence of high blood pressure in our cohort was 14.6%(11.1% with pre-hypertension and 3.5% with hypertension), a figure similar to that found in other African countries such as Egypt,^18^ South Africa,^24^ the Seychelles^26^ and Sudan,^28^ as wellas non-African countries, including Spain,^17^ United States,^1^Canada,^29^ Argentina^25^ and Brazil.^16^,^27^ A study in young adults(18 to 29 years) in Angola,^30^ however, showed a prevalenceof hypertension of 23%, suggesting a rapid increase in bloodpressure during adolescence and early adulthood

According to our findings, it is likely that body fat accumulation during adolescence may contribute to increased blood pressure, thus contributing to early onset of hypertension in adults. Validating other findings,^31^ we observed that elevated blood pressure was higher in overweight and obese children. Constanzi et al. showed that schoolchildren with increased waist circumference were 2.8 times more likely to have high blood pressure levels than children with a normal waist circumference.^27^

Hypertension is a multifactorial disease and the pathogenesis of its relationship with fat accumulation is unknown. Sorof et al. showed that obesity-induced hypertension could be due in part to hyperactivity of the sympathetic nervous system, which is manifested as increased heart rate and blood pressure variability.^32^ This association, however, was not identified in our study since the overweight/obese children showed lower heart rates at rest.

Another link between increasing blood pressure in children and fat accumulation is the increased thickness of the intima–media layer of arteries, leading to increased peripheral vascular resistance. Wunsch et al. showed an increased intima–media thickness in carotid ultrasonography of obese children. After a long period of physical activity and substantial weight loss, blood pressure reduction was followed by a proportional reduction of the carotid intima–media thickness, as well as in other blood biochemical parameters.^33^

Genetic and environmental factors have also been suggested to be linked to high blood pressure. Therefore we also investigated the association of high blood pressure in children with a familial history of hypertension. Despite the fact that 72.4% of children with high blood pressure reported a positive family history of hypertension, this association was not significant when compared with children with normal blood pressure and positive family history of the disease (χ2 = 0.247; p = 0.618). Our data are consistent with those of Shi et al.^29^ who also observed that the presence of family history of hypertension did not change the association of high blood pressure and BMI.

Overweight and obesity is a huge health problem in developed countries, and its prevalence seems to increase rapidly in countries facing fast economic growth.^23^ Another study by our group in adults (20–72 years) indicated a prevalence of overweight and obesity of 29.3 and 19.6%, respectively.^34^ The increasing incidence of obesity is worrisome, not only because it represents a disease per se but also because it is an important risk factor for the development of diabetes and cardiovascular diseases in the future.^35^

The obesity pandemic therefore predicts the rise in incidence of other diseases in the near future. For this reason, obesity should be viewed as one of the most pressing health problems faced by public health authorities this century.^23^ The prevalence of obesity, however, has shown variable prevalence in different countries and in different populations within a single country, varying from one to 20%.

In our study, we detected excessive weight in 17.7% of the studied sample, most reaching BMI values compatible with obesity, without significant gender differences. These figures are similar to studies in countries such as Egypt,^18^ the Seychelles,^26^ Sudan,^28^ Brazil^16^,^27^,^36^ and Switzerland,^37^ but differ from others studies, such as in South Africa, where children living in rural areas were studied.^24^ Urbanisation therefore seems to be an important factor facilitating the development of obesity in children. One feature that distinguishes our results from other studies was the higher prevalence of obesity than overweight. Similar results were found by Constanzi et al.^27^ in Brazilian schoolchildren with similar characteristics to those included in our study. 

The high prevalence of dyslipidaemia detected in our study is also noteworthy. Interestingly, dyslipidaemias were not associated with either fat accumulation or high blood pressure. However, the lack of a statistically significant association should be viewed with caution, given the small sample size of children showing obesity and/or hypertension. Our results suggest, however, that development of these cardiovascular risk factors may depend on different predictors. Dyslipidaemias, however, may be detected precociously because they may produce early changes in vascular structure, facilitating the development of atherosclerosis.

Studies have alerted us to the increase in incidence of dyslipidaemia in children and adolescents.^38^,^39^ No epidemiological data on the prevalence of dyslipidaemia in this age group is available at the regional or national level in Angola, but a study conducted in adults observed that 50.1% showed low HDL cholesterol levels, while 11.1% showed hypercholesterolaemia, and 10.6% hypertriglyceridaemia.^35^

The cut-off point adopted in our study, however, was used to classify children from different genetic backgrounds and with different dietary patterns. The prevalence of dyslipidaemias found in our study [50% with total cholesterol levels > 170 mg/dl (4.4 mmol/l)] is higher than the values found in some studies^38^,^41^ that used the same cut-off points. Other studies (Muscatine study and Bogalusa Heart study) showed similar values to ours,^42^ however, the cut-off points were different from those used in our study, making comparisons impractical.^42^

Regardless of cut-off points, it is important to highlight the high prevalence of dyslipidaemia in children and adolescents. For some authors, the change is due to the development of insulin resistance, initially peripheral and later at the systemic level.^43^ In our study, 16.8% of the children with dyslipidaemia also showed impaired glucose tolerance. From the fasting glycaemia determinations, we did not find diabetic children in our sample. However, an inverse relationship was observed between BMI and fasting glycaemia. This finding may be due to the hyperinsulinaemic state of children with a higher body fat accumulation.

Our study suggests that, similar to other countries in sub-Saharan Africa, the high prevalence of overweight and obesity and of other cardiovascular risk factor indicates a rapid epidemiological transition affecting not only adults but also children.^44^ Weight gain and dyslipidaemia may be associated with environmental factors related to lifestyle, including inappropriate eating habits and physical inactivity.

The consequences of childhood dyslipidaemia on the cardiovascular health of adults was reported by Berenson et al.^45^ who described a positive association between the extent of arterial intima surface covered with atherosclerotic lesions and elevated cholesterol and triglyceride levels as well as low HDL cholesterol levels. According to Freedman et al.,^46^ dyslipidaemia and overweight/obesity in childhood causes accumulation of harmful effects over time, resulting in a thickened intima–media layer of large arteries, atherosclerosis, arterial stiffness and consequent increase in blood pressure, coronary artery disease and stroke.

## Limitations

Our study has some limitations. Children from a single school were included, therefore our data cannot be translated to the overall population of Luanda. Since the school is located in a neighbourhood with families belonging to the middle class, it represents only the population attending public school with such characteristics. Moreover, the final sample (n = 198) was sufficient to determine a prevalence around 10% only, with an estimated error of 3.5% and power of 90%, making our study inadequate for subgroup analysis.

We only included pre-pubertal children in the analysis to avoid the influence of adolescence in fat accumulation. In this group, the association between body fat and blood pressure can be observed without the well-known influence of sex hormones. Therefore, most of the analysis was conducted in the overall sample, increasing the robustness of the conclusions.

Blood pressure was measured on a single day, which is insufficient to establish a diagnosis of hypertension and rather requires confirmation by additional measures. Our analysis, however, was conducted by considering blood pressure as a continuous variable.

Finally, cut-off points used in this study were based on international references not validated for the Angolan population. However, by using such international standards, comparison with other studies was possible.

## Conclusions

Our study shows a relatively high prevalence of obesity in pre-pubertal schoolchildren in Angola and suggests a possible association between fat accumulation and high blood pressure in this age group. However, a causal relationship cannot be established in this study given its transverse design. The high prevalence of dyslipidaemia in the studied population deserves attention in future studies, which should aim to investigate potential causal relationships, including eating habits and genetic factors. Early monitoring of blood pressure and lipid profile is necessary for adoption of adequate prevention policies in the area of cardiovascular health.
